# Camera trap records of leucistic Eurasian badgers (*Meles meles*) in central Norway

**DOI:** 10.1002/ece3.8052

**Published:** 2021-09-06

**Authors:** Tim R. Hofmeester, Neri H. Thorsen, John D. C. Linnell, John Odden

**Affiliations:** ^1^ Department of Wildlife, Fish, and Environmental Studies Swedish University of Agricultural Sciences Umeå Sweden; ^2^ Norwegian Institute for Nature Research Oslo Norway; ^3^ Norwegian Institute for Nature Research Trondheim Norway; ^4^ Department of Forestry and Wildlife Management Inland Norway University of Applied Sciences Koppang Norway

**Keywords:** albinism, anomalous color, Coat coloration, *Mustelidae*

## Abstract

Coat coloration plays an important role in communication, camouflage, and sexual selection in animals. Genetic mutations can lead to anomalous colorations such as melanism and leucism, where animals appear, respectively, darker or lighter than normal. Reporting abnormal coloration in wild animals is an important first step to understand the distribution, prevalence, and potential fitness consequences of these rare events. Here, we report several records of suspected leucism in the Eurasian badger (*Meles meles*) in a population in central Norway. Several camera traps recorded at least two leucistic individuals between 2017 and 2020. It took considerable effort, almost 400,000 camera trap nights over a period of 10 years all over Norway, to obtain a total of eleven records of leucistic badgers, indicating the rarity of this phenotype. It is unclear what has caused the presence of multiple leucistic badgers in a single population, but recent colonization and lack of predators might have played a role. Due to our observations, future studies can now be developed to study the underlying mechanisms and potential consequences of leucism in this badger population. The increasing use of networks of camera traps in wildlife research will provide new opportunities to record rare coloration in wild animals.

## Graphical abstract

We recorded several leucistic Eurasian badgers with camera traps in central Norway. These badgers have occurred in the area for several years, but it is unclear what has caused several leucistic badgers to occur in the area.

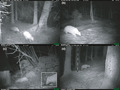

## 1 INTRODUCTION

Coat coloration plays an important adaptive role in mammals, functioning as camouflage and in both intra‐ and interspecific communication (Caro, 2005). Coloration anomalies due to a deficit or excess of the production of melanin, such as albinism, leucism, erythrism, and melanism, are relatively rare in wild mammals, with only a few records of single individuals for some species (Abreu et al., 2013; Łopucki & Mróz, 2010). Melanism is characterized by an excess of melanin resulting in a darker than normal coat coloration, while albinism and leucism are characterized by a deficit of melanin, where albinos are distinguished by the total lack of pigmentation including in the eyes, resulting in a lighter‐than‐normal coat coloration (Sage, 1962). Erythrism is also characterized by a deficit of melanin, but with an abundance of reddish color resulting in a lighter and more red appearance compared with the normal phenotype (Laacke et al., 2006). These colorations often occur in isolated and genetically homogenous populations as they are caused by single mutations in specific genes (Hubbard et al., 2010). There are some studies showing the adaptive advantage of coat color mutations (e.g., Linnen et al., 2009), but these studies are rare (Hubbard et al., 2010). This is likely due to the rarity of the occurrence of these anomalies that suggests a natural selection against these coat colorations (Caro, 2005). However, in order to better study coat color anomalies, we first need better information on how often and where these color anomalies occur in wild populations.

A recent review found 114 studies that reported leucism in wild mammals, of which 33 studies reported leucism in a species from the order Carnivora (Olson & Allen, 2019). Of these 33 studies, 18 pertained to the mustelid family (*Mustelidae*), of which 14 reported leucism in the tayra (*Eira barbara*; e.g., Sobroza et al., 2016; Talamoni et al., 2017; Scrich et al., 2019; Pontes et al., 2020). Other mustelids in which leucism or albinism has been recorded are Neotropical otter (*Lontra longicaudis*; Arriaga‐Flores et al., 2016), Eurasian otter (*Lutra lutra*; Goncharuk et al., 2020), oriental small‐clawed otter (*Aonyx cinereus*; Allen et al., 2019), and fisher (*Pekania pennanti*; Olson & Allen, 2019). Albinism has also been described in domestic mustelids, both the ferret (*Mustela putorius furo*; Syed et al., 1987), and American mink (*Neovison vison*; Anistoroaei et al., 2008). Several species of mustelids, such as the striped polecat (*Ictonyx striatus*), greater grison (*Galictis vittata*), and Eurasian badger (*Meles meles*), use distinctive coat coloration for both inter‐ and intraspecific communication (Buesching & Stankowich, 2017; Kitchener et al., 2017). Thus, coat color mutations, such as leucism, might impact these species to a greater extent than most, as not only hunting success and survival but also inter‐ and intraspecific communication might be impacted by coat color anomalies.

**FIGURE 1:**
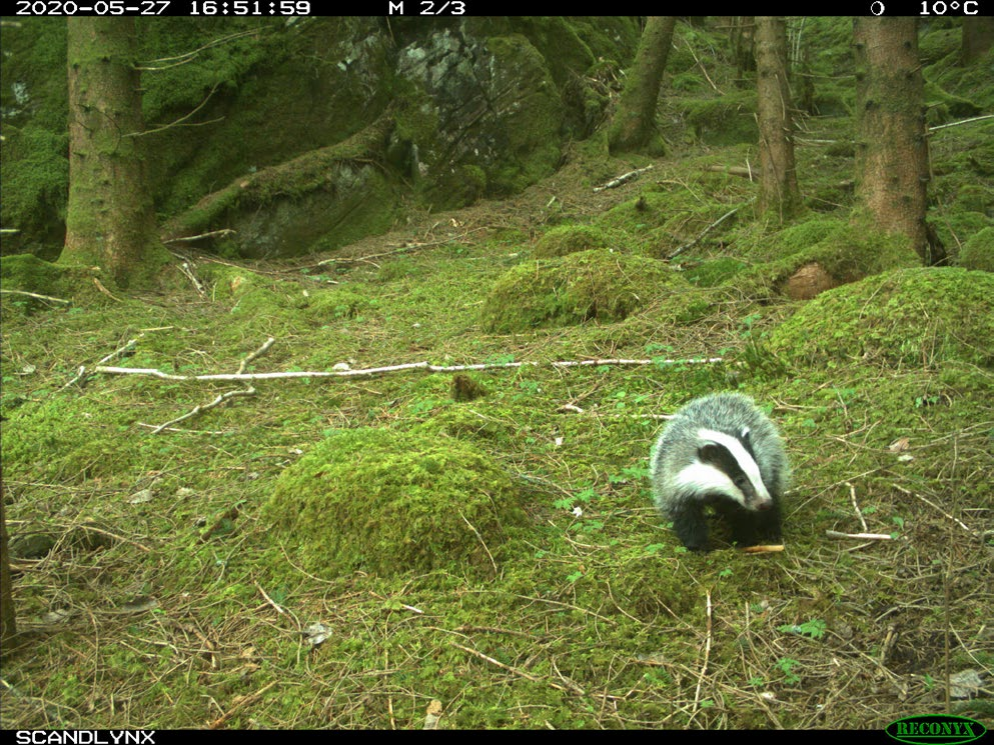
Camera trap image of a regularly colored badger from the study area, showing the distinct gray‐brown body, dark legs, and black and white facial marks

The Eurasian badger normally has a distinct coat coloration with a gray‐brown body, dark legs, and black and white marks on the head (Figure 1; Kruuk, 1989; Roper, 2010). Albino, leucistic, melanistic, and erythristic Eurasian badgers have been described before (Roper, 2010) and seem to occasionally be sighted in the UK (Badger Trust, 2021). However, to date these observations have not been recorded in an accessible format, such as in the scientific literature, making it difficult to assess the frequency of occurrence and study the causes and consequences of coat color anomalies in Eurasian badgers. Here, we report the observation of several leucistic Eurasian badgers in Norway.

## 2 METHODS

### 2.1 Study area

**FIGURE 2:**
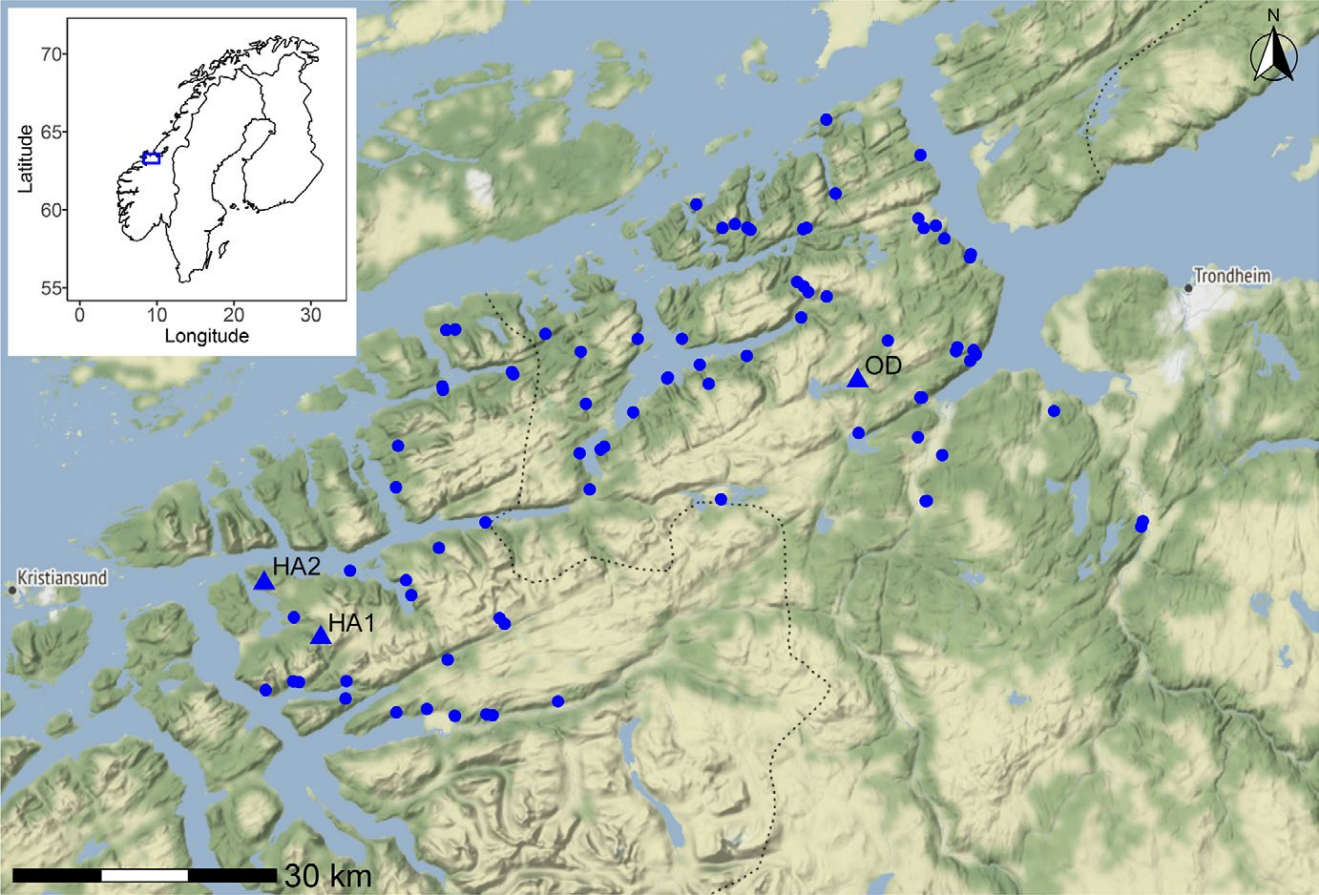
Study area where we detected the leucistic badgers. Blue points represent camera trap locations, the three locations where we detected leucistic badgers are presented as triangles: OD—the location in Orkdal; HA1 and HA2—the two locations close to Halsa. Inset: location of the study area in Norway (blue square). Background: map tiles by Stamen Design, under CC BY 3.0 Data by OpenStreetMap, under ODbL

We placed 90 camera traps (HC500, HC600, PC850, and PC900; Reconyx, Inc.) in a study area on the border of Møre og Romsdal and Trøndelag counties in central Norway (63°16′N 9°32′E; Figure 2). The study area is situated on the coast of the Atlantic Ocean and is characterized by relatively mild winters and a rugged coastline of many fjords, islands, and peninsulas. It is relatively isolated with the Atlantic coast and two large fjords on the north, northeast, and southwest sides. On the south, it is bordered by a mountain range. The topography consists of many forested hills and mountains with rocky outcrops and bare rock at higher elevations. Human activity is centered on the coast and valley bottoms, where there is some agriculture. The 90 camera trapping sites in the study area have been active since the winter of 2016–2017 and are part of several hundred, often volunteer‐run, camera trap sites distributed throughout Norway with the aim of monitoring Eurasian lynx (*Lynx lynx*) family groups within the Scandcam project (see viltkamera.nina.no for more information, locations, and photographs of recorded animals). Eurasian badgers are native to Norway and occur as far north as the Arctic Circle, but are absent from large parts of western Norway (Artsdatabanken, 2021). The study area was colonized from the east only relatively recently, likely somewhere between the 1970s and the 1990s (Bevanger & Lindström, 1995).

## 3 RESULTS

**FIGURE 3:**
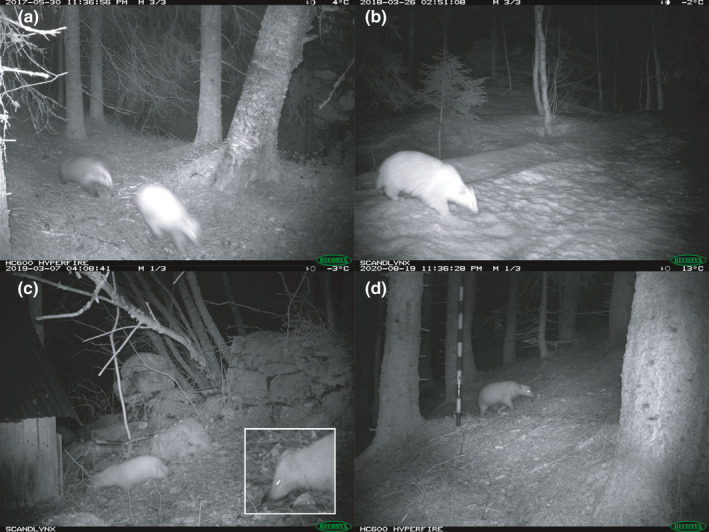
Records of leucistic Eurasian badgers (*Meles meles*) in central Norway. (a) First record of a leucistic badger in Norway. A regularly colored badger (left) chases the leucistic badger (right) showing the clear difference in coloration. (b) Second record of a leucistic badger at a different location. (c) Third record of a leucistic badger at a third location, inset: close‐up of the head showing the dark nose. (d) Last record of a leucistic badger at the same location as A but from a different camera angle

We first recorded a light‐colored badger interacting with a regularly colored badger (Figure 3a) on 30 May 2017 in a location in Orkdal (63°19′52.6″N 9°38′31.7″E, OD in Figure 2). A second record at the same site was obtained on 19 August 2020 (Figure 3d). This is potentially the same individual as badgers can reach ages of more than 3 years (Macdonald & Newman, 2002), but might also be another (related) individual. On 26 March 2018, we recorded a light‐colored badger (Figure 3b) in a location close to Halsa (63°04′04.1″N 8°25′05.2″E, HA1 in Figure 2) 68 km from the OD location. On 7 March 2019, we recorded another light‐colored badger (Figure 3c) in a second location close to Halsa (63°05′18.5″N 8°21′24.6″E, HA2 in Figure 2), 4 km from the HA1 location. We have seven more observations at the HA2 location over the period between 21 March 2019 and 18 August 2020, potentially of the same badger or several related individuals. We have never observed more than one leucistic badger at the same time and were unable to find any other distinguishing features that could help identify individuals. All observed leucistic individuals were fully grown, at least one‐year‐old badgers. However, because the two records on 18 and 19 August 2020 at OD and HA2 were 70 km apart, we conclude that we have observed at least two individuals.

We believe these to be leucistic Eurasian badgers as several images show a dark nose (inset Figure 3c). Such dark nose coloration has also previously been found in a leucistic fisher (Olson & Allen, 2019). Taking into account the total effort in the Scandcam project, 386,589 camera trap nights (approximately 1,060 camera trap years) over 10 years at 1,399 locations spread throughout Norway, we conclude that leucism is rare in Norwegian badgers. In the total dataset, we only recorded 11 observations of leucistic badgers in a total of 19,820 independent badger observations, with 2,442 observations of badgers at the 90 camera locations in the study area. We consider each sequence of images taken with a maximum time interval of 5 min between previous and subsequent images containing one or more badgers as an independent observation. If we assume an equal detectability of leucistic badgers compared with normal coloration, we derive a frequency of 0.5% leucistic badgers in the study area, and 0.06% in the whole of Norway.

## 4 DISCUSSION

Distinct coloration in the anterior parts of the body in mesocarnivores is likely related to aposematic communication toward larger predators (Buesching & Stankowich, 2017; Caro et al., 2017; Newman et al., 2005), and it has been suggested that the facial marks in Eurasian badgers might also have a function in intraspecific communication (Kitchener et al., 2017). Therefore, leucism might affect intra‐ and interspecific communication in badgers. Furthermore, leucism will increase the visibility of the badger, as it is lighter than its surroundings, which might increase predation risk, except for situations when there is snow on the ground. Only two out of 11 observations of leucistic badgers were under snowy conditions (Figure 3b). This is likely because badgers reduce body temperature and activity in winter as they have difficulty hunting when the ground is covered in snow or ice (Bevanger & Brøseth, 1998; Bevanger & Lindström, 1995; Kruuk, 1989), reducing the potential adaptive advantage of being light‐colored. Alternatively, increased visibility might potentially also decrease the risk of crossing roads, an important cause of mortality in many badger populations (Griffiths & Thomas, 1993). However, studies on the importance of different causes of mortality for badgers in Norway are lacking. Consequently, it is hard to say whether the light coloration causes an adaptive disadvantage for badgers in Norway, especially as all observed individuals at least survived to be fully grown.

Although it is unknown what has caused multiple leucistic badgers to occur in the study region, there are several factors that might have played a role. As badgers only recently colonized the study area, there might be a founder effect that caused low genetic diversity and a larger probability of rare phenotypes being expressed (Hubbard et al., 2010). Due to the topographically fragmented landscape of fjords and mountains, it is likely that only a few individuals founded the badger population in the study area, which would increase the chance of a point mutation, such as leucism, spreading through the population. A population genetic study of the badger population in the study area is, however, lacking. Furthermore, the lack of predators of adult badgers, such as wolves and wolverines (Olsson et al., 1997), in the study area likely results in a low increased mortality risk due to leucism, although it is allowed for people to hunt badgers in the study area. Lastly, it cannot be ruled out that local environmental contamination might have caused an increased presence of leucism (Møller & Mousseau, 2001). It would thus be very interesting to study the genetics of this badger population to unravel the genetic history of the leucistic badgers and determine whether the leucistic individuals are genetically related to each other. That would also allow for a better estimation of the number of leucistic individuals and the proportion of leucistic badgers in the population. Furthermore, it would enable the study of other deleterious mutation that might be associated with the presence of leucism in badgers, similar to albinism‐related diseases in humans (Witkop et al., 1990).

Leucism has been found in several other carnivore species, especially in the Neotropics, and seems to be relatively common in mustelids (Abreu et al., 2013; Olson & Allen, 2019). Similar to our observations, many of these records were from adult individuals, questioning the impact of leucism on hunting success and survival. Although many of the known records of leucism in carnivores are from animals in museum collections, an increasing number of records are being collected using camera traps (Allen et al., 2019; Cronemberger et al., 2018; Olson & Allen, 2019; Scrich et al., 2019). An advantage of the use of camera traps to study the presence and frequency of leucism in wild populations is that camera traps are not biased in reporting color anomalies, something that might impact studies based on collected specimens or sightings. Furthermore, camera traps might be used to study the presence of predators and prey of the leucistic carnivores, making it possible to infer how hunting success and survival might differ in populations with leucistic individuals compared to populations without. Lastly, camera traps can be used to monitor badger sets to study the number of offspring and offspring survival of sets with or without leucistic badgers, to derive estimates of fitness consequences of fur coloration (Bernardi et al., 2021).

As there is little knowledge about the causes and consequences of color anomalies in wild mammals, it is important to get a better understanding of where and how often these anomalies occur (Caro, 2005; Łopucki & Mróz, 2010). Only then can we study the locations where the anomalies occur to get a better understanding of the underlying mechanisms and potential fitness consequences. As the number of camera traps deployed by scientists, conservationists, and the general public is increasing (Burton et al., 2015; Rowcliffe & Carbone, 2008), these devices will pick up more and more rare events, such as color anomalies and rare behaviors (Cove et al., 2017). It is important to report these rare events to build up a larger number of scientific publications of color anomalies in wild mammals to better understand how often these anomalies occur and to provide a starting point for further studies.

## CONFLICT OF INTEREST

The authors declare no competing interests.

## AUTHOR CONTRIBUTIONS

**Tim R. Hofmeester:** Conceptualization (lead); formal analysis (lead); funding acquisition (equal); investigation (equal); methodology (equal); visualization (lead); writing—original draft (lead); writing—review and editing (lead). **Neri H. Thorsen:** Data curation (equal); investigation (equal); methodology (equal); writing—review and editing (equal). **John D. C. Linnell:** Conceptualization (equal); funding acquisition (equal); investigation (equal); methodology (equal); writing—review and editing (equal). **John Odden:** Conceptualization (equal); data curation (equal); funding acquisition (lead); investigation (equal); methodology (equal); project administration (lead); writing—review and editing (equal).

## Data Availability

All camera trap data from the Scandcam project (locations and images) are available on viltkamera.nina.no.

## References

[ece38052-bib-0001] Abreu, M. , Machado, R. , Barbieri, F. , Freitas, N. S. , & Oliveira, L. R. (2013). Anomalous colour in Neotropical mammals: A review with new records for *Didelphis* sp (Didelphidae, Didelphimorphia) and *Arctocephalus* *australis* (Otariidae, Carnivora). Brazilian Journal of Biology, 73, 185–194. 10.1590/S1519-69842013000100020 23644801

[ece38052-bib-0002] Allen, M. L. , Sibarani, M. C. , & Utoyo, L. (2019). The first record of a wild hypopigmented Oriental small‐clawed otter (*Aonyx* *cinereus*). International Journal, 21, 201904.

[ece38052-bib-0003] Anistoroaei, R. , Fredholm, M. , Christensen, K. , & Leeb, T. (2008). Albinism in the American mink (*Neovison* *vison*) is associated with a tyrosinase nonsense mutation. Animal Genetics, 39, 645–648. 10.1111/j.1365-2052.2008.01788.x 18822100

[ece38052-bib-0004] Arriaga‐Flores, J. C. , Rodríguez‐Ruíz, E. R. , Gallo‐Reynoso, J. P. , & Castro‐Arellano, I. (2016). Leucism in neotropical otters (*Lontra* *longicaudis* *annectens*) from Mexico. The Southwestern Naturalist, 61, 63–68. 10.1894/0038-4909-61.1.63

[ece38052-bib-0005] Artsdatabanken (2021). Artskart ‐ Grevling. https://artskart.artsdatabanken.no/app/#map/427864,7623020/3/background/greyMap/filter/%7B%22TaxonIds%22%3A%5B31217%5D%2C%22IncludeSubTaxonIds%22%3Atrue%2C%22Found%22%3A%5B2%5D%2C%22NotRecovered%22%3A%5B2%5D%2C%22Style%22%3A1%7D. Accessed August 5, 2021.

[ece38052-bib-0006] Badger Trust (2021). Badger colouring. Badger Trust. https://www.badgertrust.org.uk/badgers

[ece38052-bib-0007] Bevanger, K. , & Brøseth, H. (1998). Body temperature changes in wild‐living badgers *Meles* *meles* through the winter. Wildlife Biology, 4, 97–101. 10.2981/wlb.1998.006

[ece38052-bib-0008] Bevanger, K. , & Lindström, E. R. (1995). Distributional history of the European badger *Meles* *meles* in Scandinavia during the 20th century. Annales Zoologici Fennici, 32, 5–9.

[ece38052-bib-0009] Buesching, C. D. , & Stankowich, T. (2017). Communication amongst the musteloids: Signs, signals, and cues. In D. W. Macdonald , C. Newman & L. A. Harrington (Eds.), Biology and conservation of musteloids (1st ed.., pp. 149–166). Oxford University Press.

[ece38052-bib-0010] Burton, A. C. , Neilson, E. , Moreira, D. , Ladle, A. , Steenweg, R. , Fisher, J. T. , Bayne, E. , & Boutin, S. (2015). Wildlife camera trapping: A review and recommendations for linking surveys to ecological processes. Journal of Applied Ecology, 52, 675–685. 10.1111/1365-2664.12432

[ece38052-bib-0011] Caro, T. (2005). The adaptive significance of coloration in mammals. BioScience, 55, 125–136.

[ece38052-bib-0012] Caro, T. , Walker, H. , Santana, S. E. , & Stankowich, T. (2017). The evolution of anterior coloration in carnivorans. Behavioral Ecology and Sociobiology, 71, 177. 10.1007/s00265-017-2402-5

[ece38052-bib-0013] Cove, M. V. , Maurer, A. S. , & O’Connell, A. F. (2017). Camera traps reveal an apparent mutualism between a common mesocarnivore and an endangered ungulate. Mammalian Biology, 87, 143–145. 10.1016/j.mambio.2017.08.007

[ece38052-bib-0014] Cronemberger, C. , De Aguiar, P. F. , De Faria Bacellar, A. E. , & Gonçalves Da Silva, L. (2018). First record of leucism in puma from Serra dos Órgãos National Park, Brazil. Cat News, 68, 38–40. 10.17605/OSF.IO/FY9A3

[ece38052-bib-0015] Di Bernardi, C. , Thierry, A.‐M. , Eide, N. E. , Bowler, D. E. , Rød‐Eriksen, L. , Blumentrath, S. , Tietgen, L. , Sandercock, B. K. , Flagstad, Ø. , & Landa, A. (2021). Fitness and fur colouration: Testing the camouflage and thermoregulation hypotheses in an Arctic mammal. Journal of Animal Ecology, 90, 1328–1340. 10.1111/1365-2656.13457 33660289

[ece38052-bib-0016] Goncharuk, M. S. , Voloshina, I. V. , Aramilev, V. V. , Shurygina, A. A. , & Kerley, L. L. (2020). Recent observations of Eurasian otter *Lutra* *lutra*, including white‐coated individuals, in the southern Sikhote Alin, the Russian far east. IUCN Otter Specialist Group Bulletin, 37, 147–157.

[ece38052-bib-0017] Griffiths, H. I. , & Thomas, D. H. (1993). The status of the Badger *Meles* *meles* (L., 1758) (Carnivora, Mustelidae) in Europe. Mammal Review, 23, 17–58. 10.1111/j.1365-2907.1993.tb00415.x

[ece38052-bib-0018] Hubbard, J. K. , Uy, J. A. C. , Hauber, M. E. , Hoekstra, H. E. , & Safran, R. J. (2010). Vertebrate pigmentation: From underlying genes to adaptive function. Trends in Genetics, 26, 231–239. 10.1016/j.tig.2010.02.002 20381892

[ece38052-bib-0019] Kitchener, A. C. , Meloro, C. , & Williams, T. M. (2017). Form and function in the musteloids. In D. W. Macdonald , C. Newman , & L. A. Harrington (Eds.), Biology and conservation of musteloids (1st ed., pp. 92–128). Oxford University Press.

[ece38052-bib-0020] Kruuk, H. (1989). The social badger: Ecology and behaviour of a group‐living carnivore (*Meles* *meles*). Oxford University Press.

[ece38052-bib-0021] Laacke, R. J. , Laudenslayer, W. F. , Diamond, T. , Viotto, K. , & Long, C. A. (2006). Erythrism in the North American Badger, *Taxidea* *taxus* . The Southwestern Naturalist, 51, 289–291.

[ece38052-bib-0022] Linnen, C. R. , Kingsley, E. P. , Jensen, J. D. , & Hoekstra, H. E. (2009). On the origin and spread of an adaptive allele in deer mice. Science, 325, 1095–1098. 10.1126/science.1175826 19713521 PMC2736094

[ece38052-bib-0023] Łopucki, R. , & Mróz, I. (2010). Cases of colouration anomalies in small mammals in Poland, and reasons for their incidence. Annales Universitatis Mariae Curie‐Skłodowska Lublin ‐ Polonia, 55, 67–76.

[ece38052-bib-0024] Macdonald, D. W. , & Newman, C. (2002). Population dynamics of badgers (*Meles* *meles*) in Oxfordshire, U.K.: Numbers, density and cohort life histories, and a possible role of climate change in population growth. Journal of Zoology, 256, 121–138. 10.1017/S0952836902000158

[ece38052-bib-0025] Møller, A. P. , & Mousseau, T. A. (2001). Albinism and phenotype of barn swallows (*Hirundo* *rustica*) from Chernobyl. Evolution, 55, 2097–2104. 10.1111/j.0014-3820.2001.tb01324.x 11761068

[ece38052-bib-0026] Newman, C. , Buesching, C. D. , & Wolff, J. O. (2005). The function of facial masks in “Midguild” carnivores. Oikos, 108, 623–633. 10.1111/j.0030-1299.2005.13399.x

[ece38052-bib-0027] Olson, L. O. , & Allen, M. L. (2019). A Leucisitic Fisher (*Pekania* *pennanti*) and the Prevalence of Leucism in Wild Carnivores. The American Midland Naturalist, 181(1), 133–138. 10.1674/0003-0031-181.1.133

[ece38052-bib-0028] Olsson, O. , Wirtberg, J. , Andersson, M. , & Wirtberg, I. (1997). Wolf *Canis* *lupus* predation on moose *Alces* *alces* and roe deer *Capreolus* *capreolus* in south‐central Scandinavia. Wildlife Biology, 3, 13–25. 10.2981/wlb.1997.003

[ece38052-bib-0029] Pontes, A. R. M. , Júnior, A. P. D. S. , & Chivers, D. (2020). The occurrence of leucism in groups of tayras *Eira* *barbara* Linnaeus 1758 on the Guyana shield. Écoscience, 27, 295–301. 10.1080/11956860.2020.1804724

[ece38052-bib-0030] Roper, T. J. (2010). Badger. HarperCollins Publishers Limited.

[ece38052-bib-0031] Rowcliffe, J. M. , & Carbone, C. (2008). Surveys using camera traps: Are we looking to a brighter future? Animal Conservation, 11, 185–186. 10.1111/j.1469-1795.2008.00180.x

[ece38052-bib-0032] Sage, B. L. (1962). Albinism and melanism in birds. British Birds, 55, 201–225.

[ece38052-bib-0033] Scrich, V. M. , Ponzio, M. C. , Pasqualotto, N. , Rodrigues, T. F. , & Paolino, R. M. (2019). Occurrence of tayras (*Eira* *barbara* Linnaeus, 1758) with anomalous coloration in Cerrado remnants in the state of Sao Paulo, Brazil. Biota Neotropica, 19, e20180680. 10.1590/1676-0611-BN-2018-0680

[ece38052-bib-0034] Sobroza, T. V. , Gonçalves, A. L. , & dos Santos, L. S. (2016). Predation attempt and abnormal coat coloration of the tayra (*Eira* *barbara*) in the Brazilian Central Amazon. Studies on Neotropical Fauna and Environment, 51, 231–234. 10.1080/01650521.2016.1227137

[ece38052-bib-0035] Syed, M. , R⊘nningen, K. , & Nes, N. N. (1987). Inheritance of coat colour in ferrets. Acta Agriculturae Scandinavica, 37, 85–88. 10.1080/00015128709436545

[ece38052-bib-0036] Talamoni, S. , Viana, P. I. M. , Costa, C. G. , Palú, L. , Oliveira, R. B. , & Pessôa, L. M. (2017). Occurrence of leucism in *Eira* *barbara* (Carnivora, Mustelidae) in Brazil. Biota Neotropica, 17, e20170328. 10.1590/1676-0611-BN-2017-0328

[ece38052-bib-0037] Witkop, C. J. , Nuñez Babcock, M. , Rao, G. H. , Gaudier, F. , Summers, C. G. , Shanahan, F. , Harmon, K. R. , Townsend, D. , Sedano, H. O. , & King, R. A. (1990). Albinism and Hermansky‐Pudlak syndrome in Puerto Rico. Boletin De La Asociacion Medica De Puerto Rico, 82, 333–339. 2261023

